# Negative linear compressibility at extreme pressure

**DOI:** 10.1107/S2052252522001312

**Published:** 2022-02-12

**Authors:** Kamil F. Dziubek

**Affiliations:** a LENS - European Laboratory for Non-Linear Spectroscopy, Via Nello Carrara 1, 50019 Sesto Fiorentino (FI), Italy

**Keywords:** high-pressure research, negative linear compressibility, extreme conditions

## Abstract

Structural studies of chemical elements in extreme pressure conditions often lead to unpredictable and surprising results. At ultra-high pressure Yuan *et al.* [ *IUCrJ* (2022), **9**, 253–260] report a new crystal phase of selenium that exhibits negative linear compressibility.

Although the term allotropy, which signifies the ability of chemical elements to exist in different structural modifications, was introduced as early as 1840 by Jöns Jakob Berzelius (Berzelius 1841 ▸; Jensen, 2006 ▸), the number of known elements that existed in more than one polymorph was quite limited at this time. It was only later that studies under various pressure and temperature conditions became possible with the development of high-pressure experimental techniques, sparking a rash of newly discovered crystal forms, often exhibiting unusual structures and properties (Young, 1991 ▸; Tonkov & Ponyatovsky, 2005 ▸).

While many elements have been thoroughly studied under non-ambient conditions, there are still many controversies regarding their phase diagrams. In particular, phase boundaries in the pressure–temperature diagram, the sequence of phase transitions and the structure of particular phases often become the subject of debate. Inconsistencies and contradictory results concern mainly extreme pressures, where the minute size of the sample, substantial nonhydro­static stress and other factors, like kinetic effects, may significantly affect the results. With the advent of third- and fourth-generation synchrotrons, the X-ray beam is focused down to micron on even submicron size enhancing spatial resolution. In addition, the development of detectors with improved signal-to-noise ratio and fast readout has further enhanced the capabilities of the instruments. For this reason, numerous early studies have been challenged and revised recently.

Systematic investigation of group 16 elements is fascinating on many levels. While the lighter elements – oxygen and sulfur – form molecular crystals, and polonium, the heaviest element of the group observed in nature, is metallic, both selenium and tellurium are semiconductors. As a consequence, elemental selenium found numerous applications ranging from photocopying (Chou *et al.*, 2003 ▸) to flat-panel X-ray detectors (Huang & Abbaszadeh, 2020 ▸). Understanding the behaviour of compressed selenium is therefore of great interest for both fundamental physics and industrial applications. Below 60 GPa selenium exists in four various crystal phases. According to Akahama *et al.* (2021 ▸), the rhombohedral phase named Se-V (space group *R* 3 *m*, β-Po structure) occurs between ∼60 GPa and 140 GPa, where it transforms to the Se-VI b.c.c. phase, stable up to at least 317 GPa.

In this issue of **IUCrJ** , Yuan *et al.* (2022 ▸) revised the extreme pressure phase transitions in selenium and reported a hitherto unknown phase, characterized by an anomalous linear expansion in one principal direction under hydro­static pressure. This finding was possible only thanks to the state-of-the-art data collection technique. The sample was subjected to constant compression and short-time exposure using a fast detector. In this way, the authors collected more than 4300 diffraction images from ambient pressure to 210 GPa, in fine pressure steps. Careful analysis of the results revealed that the variation of Se-V lattice parameter *a* with pressure changes the slope from decreasing to increasing at 120 GPa. This trend is maintained until 148 GPa, and the Bragg peaks of the rhombohedral phase are visible up to 160 GPa.

Negative linear compressibility (NLC) is a rare phenomenon where a crystal expands along one direction under hydro­static compression (Cairns & Goodwin, 2015 ▸). It has been revealed in many classes of materials, ranging from inorganic oxides and salts to metal-organic frameworks and molecular crystals. Two of the very few examples of NLC among chemical elements are the trigonal ambient-condition polymorphs of selenium and tellurium (space group *P*3_1_21), Se-I and Te-I (Keller *et al.*, 1977 ▸). In these isostructural phases, atoms are covalently bonded in helical structures, which interact with one another through weak intermolecular forces. External pressure increase reduces interhelical distances while slightly increasing the intrinsic pitch of helices. In contrast, in Se-V each selenium atom is involved in a three-dimensional covalently bonded framework with six nearest neighbours within bonding distance (Fig. 1 ▸). This Se–Se bond is much less compressible than the second-nearest neighbour contacts, which in consequence leads to structural rearrangements and the expansion of lattice parameter *a* with pressure above 120 GPa. Cairns & Goodwin (2015 ▸) attributed such a microscopic mechanism of NLC to correlated polyhedral tilts. Noteworthy, the absolute value of NLC in Se-V is around one order smaller than that of Se-I. Hence, in one simple material, two different phases exhibit NLC in various pressure range, and in each case the mechanism can be rationalized differently at the atomic level.

Yuan *et al.* (2022 ▸) reported the new phase of elemental selenium, isostructural to Se-V, but with different properties demonstrated by its NLC. To distinguish these structures they suggested the name Se-V′ for rhombohedral selenium above 120 GPa, the pressure at which the parameter *a* begins to increase. It should be emphasized, however, that this notable discovery of NLC above one megabar could not have been accomplished without modern synchrotron radiation facilities and cutting-edge experimental techniques. Constantly evolving innovations and solutions stimulate high-pressure researchers to come up with even more daring ideas. After all, matter under extreme conditions still has many secrets to be discovered.

## Figures and Tables

**Figure 1:**
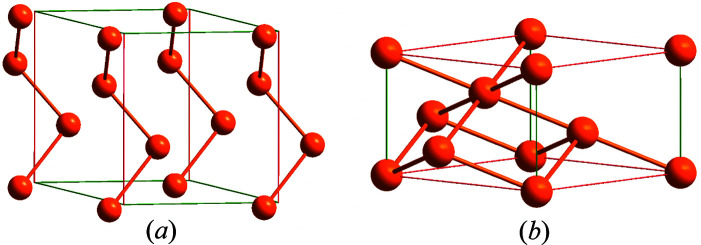
The crystal structures of Se-I (*a*) and Se-V′ (*b*). Unit-cell edges in the directions that contract with increasing pressure are shown in green, while those in the directions that expand with increasing pressure are shown in red.

## References

[bb1] Akahama, Y., Kamiue, K., Okawa, N., Kawaguchi, S., Hirao, N. & Ohishi, Y. (2021). *J. Appl. Phys.* **129**, 025901.

[bb2] Berzelius, J. J. (1841). *Årsberättelse om Framstegen i Fysik och Kemi afgifven den 31 Mars 1840. Första delen.* (*Annual Report on Progress in Physics and Chemistry submitted on 31 March 1840. First part*). Stockholm, Sweden: P. A. Norstedt & Söner.

[bb3] Cairns, A. B. & Goodwin, A. L. (2015). *Phys. Chem. Chem. Phys.* **17**, 20449–20465. 10.1039/c5cp00442j 26019018

[bb4] Chou, J.-C., Yang, S.-Y. & Wang, Y.-S. (2003). *Mater. Chem. Phys.* **78**, 666–669.

[bb5] Huang, H. & Abbaszadeh, S. (2020). *IEEE Sens. J.* **20**, 1694–1704.

[bb6] Jensen, W. V. (2006). *J. Chem. Educ.* **83**, 838–839.

[bb7] Keller, R., Holzapfel, W. B. & Schulz, H. (1977). *Phys. Rev. B*, **16**, 4404–4412.

[bb8] Tonkov, E. Yu. & Ponyatovsky, E. G. (2005). *Phase Transformations of Elements Under High Pressure.* Boca Raton: CRC Press.

[bb9] Young, D. A. (1991). *Phase Diagrams of the Elements.* Berkeley: University of California Press.

[bb10] Yuan, S., Wang, L., Zhu, S., Liu, F., Zhang, D., Prakapenka, V. B., Tkachev, S. & Liu, H. (2022). *IUCrJ*, **9**, 253–260. 10.1107/S2052252522000252 PMC8895011 35371496

